# Concomitant Fractional Anisotropy and Volumetric Abnormalities in Temporal Lobe Epilepsy: Cross-Sectional Evidence for Progressive Neurologic Injury

**DOI:** 10.1371/journal.pone.0046791

**Published:** 2012-10-11

**Authors:** Simon S. Keller, Jan-Christoph Schoene-Bake, Jan S. Gerdes, Bernd Weber, Michael Deppe

**Affiliations:** 1 Department of Neurology, University of Münster, Münster, Germany; 2 Department of Clinical Neuroscience, Institute of Psychiatry, King's College London, London, United Kingdom; 3 Department of Epileptology, University of Bonn, Bonn, Germany; 4 Department of NeuroCognition/Imaging, Life&Brain Research Center, Bonn, Germany; University of Maryland, College Park, United States of America

## Abstract

**Background:**

In patients with temporal lobe epilepsy and associated hippocampal sclerosis (TLEhs) there are brain abnormalities extending beyond the presumed epileptogenic zone as revealed separately in conventional magnetic resonance imaging (MRI) and MR diffusion tensor imaging (DTI) studies. However, little is known about the relation between macroscopic atrophy (revealed by volumetric MRI) and microstructural degeneration (inferred by DTI).

**Methodology/Principal Findings:**

For 62 patients with unilateral TLEhs and 68 healthy controls, we determined volumes and mean fractional anisotropy (FA) of ipsilateral and contralateral brain structures from T1-weighted and DTI data, respectively. We report significant volume atrophy and FA alterations of temporal lobe, subcortical and callosal regions, which were more diffuse and bilateral in patients with left TLEhs relative to right TLEhs. We observed significant relationships between volume loss and mean FA, particularly of the thalamus and putamen bilaterally. When corrected for age, duration of epilepsy was significantly correlated with FA loss of an anatomically plausible route - including ipsilateral parahippocampal gyrus and temporal lobe white matter, the thalamus bilaterally, and posterior regions of the corpus callosum that contain temporal lobe fibres - that may be suggestive of progressive brain degeneration in response to recurrent seizures.

**Conclusions/Significance:**

Chronic TLEhs is associated with interrelated DTI-derived and volume-derived brain degenerative abnormalities that are influenced by the duration of the disorder and the side of seizure onset. This work confirms previously contradictory findings by employing multi-modal imaging techniques in parallel in a large sample of patients.

## Introduction

There has been growing evidence of widespread extrahippocampal cortical and subcortical brain abnormalities in patients with temporal lobe epilepsy with associated hippocampal sclerosis (TLEhs) using quantitative imaging techniques. Most commonly, imaging studies indicate network abnormalities of temporal lobe and extratemporal lobe structures ipsilateral to TLEhs. Of particular vulnerability appear to be the integrity and structure of the ipsilateral extrahippocampal mesial temporal lobe, ipsilateral temporal lobe white matter, corpus callosum and thalamus [1], [2], [3], [4]. However, there are other regions that appear to be susceptible to structural alterations, including the frontal lobes and basal ganglia [1], [5], [6]. Inter-study differences in the distribution and extent of these alterations are most likely due to methodological factors, in particular the biological characteristic being quantified (e.g. volume atrophy, cortical thinning, white matter diffusion alterations), modality of neuroimaging investigation (e.g. T1-weighted MRI or DTI) or approach taken to quantify brain alterations (e.g. region-of-interest (ROI) or whole brain approaches). A water diffusion sensitive MR technique like DTI can potentially detect architectural alterations that appear normal on clinical MRI sequences. DTI-determined alterations in fractional anisotropy (FA) or mean diffusivity (MD) have been demonstrated in TLEhs in regions within and beyond the hippocampus [3], [4], and in MRI-negative epilepsy disorders [7], [8], [9]. Furthermore, it has been shown that neuropsychological deficits in neurological patients correlate better with alterations of FA than lesions observed on conventional MRI [10], and that alterations of FA have a histopathological basis in patients with TLEhs [11]. However, there is little information on the relationship between DTI-derived structural alterations and macroscopic gross (volumetric) atrophy of the same structures in patients with epilepsy. In the present study, we sought to directly compare regional gross anatomical atrophy and FA abnormalities - a proxy for microstructural degeneration - in patients with unilateral TLEhs.

Whether recurrent seizures cause progressive brain damage is a contentious issue [12], [13], [14], [15]. This issue is best investigated using longitudinal DTI in newly diagnosed patients, as this approach can provide important insight into relations between individual clinical and biological variables and brain microstructure at the earliest opportunity. However, there is very little work in this area, which is a program of research that is required [16]. The lack of comprehensive research in this area is probably due to the difficulty in recruiting a large enough sample of newly diagnosed patients - probably in the hundreds - to compensate for the expected high attrition rates. It is therefore necessary to use cross-sectional approaches to investigate how age of onset and duration of chronic TLE are related to brain structure and integrity, as a proxy for seizure-induced damage. Using this approach, some cross-sectional conventional MRI studies have reported correlations between duration of TLE and grey matter atrophy [17], [18], while others have not [19]. It may be expected that associations between regional brain architecture and clinical variables would be more revealing using also DTI, given the additional sensitivity of DTI in the detection of pathological microstructural brain alterations. However, there are only few DTI studies investigating such associations, and in those that have, patient numbers have been too small to generate statistically meaningful results. DTI studies of TLEhs reporting (typically no or minimal) associations between FA and clinical variables have included samples of between nine [20] and 35 [4] patients with TLEhs. In the present study, we sought to investigate the effects of duration of TLEhs on brain structural degeneration in a relatively large sample of patients with TLEhs, with simultaneous consideration of rates of degeneration due to normal aging.

TLEhs occurs more frequently in the left hemisphere relative to the right hemisphere, a population laterality difference that is statistically significant [21]. The reasons for this are not well understood. Some have suggested that this laterality difference may be due to developmental factors, insomuch that the slower and later maturation of the left hemisphere may leave ipsilateral structures more susceptible over a longer period of time to early neurological insults that act as initial precipitating factors in the development of TLE [22], [23]. Furthermore, the left hemisphere has been reported to be more vulnerable to hypoxic-ischemic insults given the perinatal vascular asymmetry of the two hemispheres [24]. The hippocampus is particularly vulnerable to hypoxic-ischemic insults [25]. Regardless of the aetiology of the differences in lateralization, there is a growing body of literature indicating that patients with left and right TLEhs have differing neuropathological profiles, primarily manifested as a more diffuse and bilateral distribution of brain alterations in patients with left TLEhs relative to right TLEhs [22], [26], [27], [28]. In the present study, we sought to prospectively investigate the effects of side of TLEhs on regional volume and FA alterations of structures known to be important in TLEhs.

Previous studies investigating the relationship between brain structure and TLEhs in relatively large sample sizes have been restricted to the analysis of T1-weighted MRI [12], [17], [19], [26]. The number of patients investigated in DTI studies of TLEhs is much smaller, and studies simultaneously assessing T1-weighted MRI and DTI are smaller still. In the present study, we sought to prospectively recruit a relatively large sample of consecutive patients with unilateral TLEhs and no additional pathology for multi-modal quantitative neuroimaging with the objective of investigating inter-relationships between conventional T1-weighted MRI and DTI characterised brain alterations. In doing this we were also able to investigate the effects of clinical variables and side of TLEhs on brain structure and integrity.

## Methods

### Participants

We recruited 62 consecutive patients (mean age 41.4 yrs, SD 13.6) with clinical evidence of TLE and neuroradiological evidence of unilateral hippocampal sclerosis ipsilateral to the side of seizure onset. This included 41 patients with left TLEhs and 21 patients with right TLEhs (see Table 1 for a summary of participant demographics and clinical information). There were no significance differences between patients with left and right TLEhs in age (t = 0.78, p = 0.44), age of onset of epilepsy (t = 0.07, p = 0.94) and duration of epilepsy (t = 0.81, 0.42). All patients underwent comprehensive routine pre-surgical evaluation for medically intractable TLE at the Department for Epileptology, University of Bonn. This included detailed seizure semiology, interictal EEG, long-term video EEG monitoring, epilepsy-tailored clinical MRI sequences (T1-weighted, T2-weighted and FLAIR), and neuropsychological assessment. All patients had complex partial seizures of unilateral temporal lobe origin, and no evidence of dual pathology. We also recruited 68 neurologically and psychiatrically healthy control participants who were prospectively enrolled in this study as an age- and sex-matched reference cohort (Table 1: all patients = mean age 41 years, 33 females, 29 males; controls = mean age 40 years, 35 females, 33 males). All patients and controls provided written informed consent and the ethics committees of the Universities of Bonn and Münster approved this study.

**Table 1:** Summary of demographic and clinical information of participants.

	Left TLEhs	Right TLEhs	Controls
n	41	21	68
age (years)*mean (SD)*	42 (13)	40 (15)	40 (14)
F/M	26/15	7/14	35/33
age of onset (years)*mean (SD)*	17 (11)	17 (12)	-
duration (years)*mean (SD)*	27 (14)	26 (12)	-
febrile convulsions (n/y/uk)	20/19/2	13/5/3	-

Abbreviations: n, no; SD, standard deviation; uk, unknown; y, yes.

### Image acquisition

All 130 participants underwent MR imaging at the Life & Brain Center in Bonn on a 3 Tesla scanner (Magnetom Trio, Siemens, Erlangen, Germany). All subjects underwent the same imaging protocol consisting of whole brain T1-weighted, T2-weighted (not analysed in this paper) and diffusion-weighted structural imaging. Diffusion-weighted data was obtained using a diffusion-weighted single shot spin-echo EPI sequence (TR = 12 s, TE = 100 ms, 72 axial slices, resolution 1.72×1.72×1.7 mm, no cardiac gating, GRAPPA acceleration factor 2.0). Diffusion gradients were equally distributed along 60 directions (b-value = 1000 s/mm^2^). Additionally, seven data sets with no diffusion weighting (b-value = 0 s/mm^2^) were acquired initially and interleaved after each block of 10 diffusion-weighted images. T1-weighted images were obtained using an MPRAGE sequence with 160 slices (TR = 1300 ms, TI = 650 ms, TE = 3.97 ms, resolution 1.0×1.0×1.0 mm, flip angle 10°).

### Image analysis: DTI

**Figure 1:**
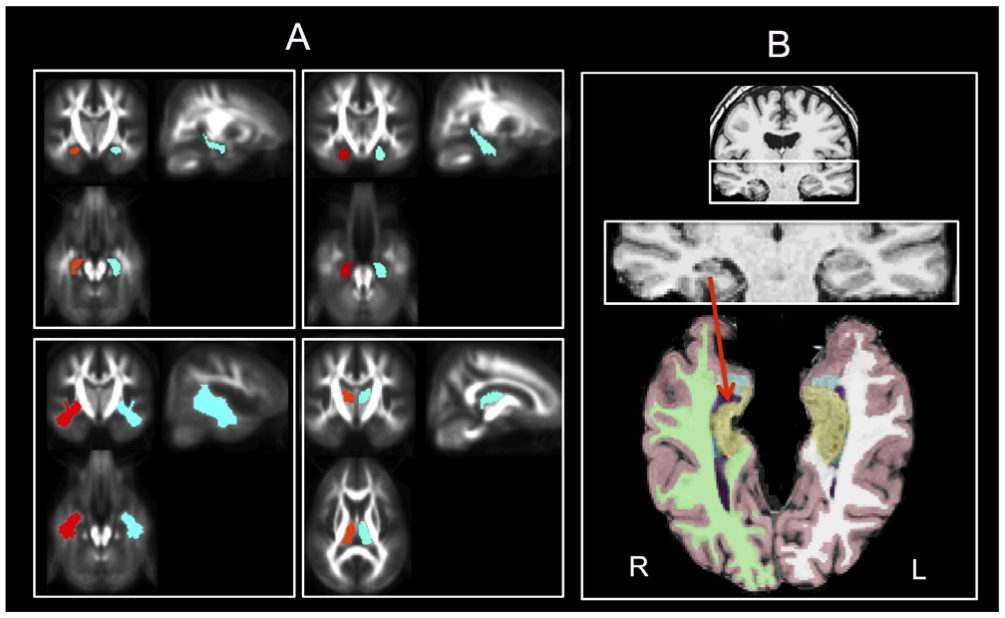
DTI and volumetric ROI approaches. A. Masks used for calculation of ROI mean FA. Examples shown are hippocampus (i), parahippocampal gyrus (ii), temporal lobe white matter (iii), and thalamus (iv). B. Automated labelling and volume estimation using FreeSurfer in a patient with right TLEhs. A supratentorial axial view (with infratentorial brain removed) is used to emphasize right hippocampal atrophy (red arrow) in this case. Yellow = hippocampus, blue = amygdala, purple = lateral ventricle, green/white = right/left cerebral white matter, pink = cortex. R, right. L, left.

All DTI image processing was performed with the “Münster Neuroimaging Evaluation System (EVAL)” employing a multi-contrast image registration toolbox that was developed for optimal image processing for FA images and a recently developed 3D eddy current correction approach running on a 64-processor parallel computer (Sun Microsystems, Inc., Palo Alto). Detailed descriptions of the DTI post-processing used in the present study can be found in Mohammadi et al. [29], which was also used in our recent epilepsy studies [8], [9]. Numerous measures of diffusion and its spatial anisotropy have been proposed, which could lead to confusion in interpreting and comparing results from different studies [30]. Here we decided to assess DTI-derived brain alterations *only* by FA, because we wanted to focus our results on a robust and commonly used scalar measure so that the results remain structured and clear. We have previously found FA a more sensitive marker of brain pathology relative to MD in patients with TLE [9] and correlations between *in-vivo* DTI and histological assessment of resected temporal lobe specimens has only been reported for FA in patients with epilepsy [11]. We obtained mean regional FA values from ROI masks that were *a-priori*-defined in MNI space to enable us to quantify the degree to which FA was altered in patients in selected brain regions [8]. Mean FA of larger brain regions including temporal lobe white matter, frontal lobe white matter, corpus callosum (and genu and splenium separately), internal capsules, and brainstem were calculated, based on the previously reported diffusivity abnormalities of these regions in patients with TLEhs [3], [4]. We also calculated mean FA of the left and right hippocampus, parahippocampal gyrus, putamen and thalamus given the known susceptibility of these regions in TLEhs [1], [6]. Examples of some of these regions are shown in Figure 1A.

### Image analysis: MRI

All quantitative MRI analyses were performed on T1-weighted MPRAGE images using an Apple® Mac Pro (OS X 10.7, 32 GB, 2×2.93 GHz 6-Core Intel Xeon (HT), Apple® Inc., Cupertino, CA, USA). FreeSurfer software (http://surfer.nmr.mgh.harvard.edu/) was used to obtain subcortical and cortex volumes for all patients and controls using an observer-independent approach. The volumes analysed were the left and right hippocampus, thalamus, putamen, temporal horn of the lateral ventricle and entire hemispheric cortex. We also analysed the areas of the (i) anterior, (ii) mid-anterior, (iii) central, (iv) mid-posterior and (v) posterior corpus callosum. These volumetric ROIs were selected for investigation in order to maximise correspondence with subcortical DTI ROIs. All segmentations were based on the assignment of neuroanatomical labels to each voxel in an MR image based on the probabilistic information automatically estimated from a manually labelled training set. The methods of the automated volumetric approach have been described in detail previously [31], and the accuracy of automated labelling and volumetry of subcortical structures have been independently validated with respect to ‘gold standard’ manual volumetric techniques, predominantly for the hippocampus [32], [33], [34], and also of the amygdala [33], [34], striatum [33] and thalamus [35]. The FreeSurfer ‘recon-all’ function (for cortical reconstruction and brain segmentation) segmented and labelled 23 MRIs in approximately 17 hours using the Mac Pro. After the ‘recon-all’ function, the neuroanatomical labels were inspected for accuracy in all patients and controls. Despite that FreeSurfer permits manual editing to improve subcortical segmentation, no obvious errors in the automatic labelling were observed for any subject, and so all data obtained from FreeSurfer analyses were 100% automated. Figure 1B shows a randomly selected patient with TLEhs for whom volumes have been automatically labelled and extracted.

### Statistical analysis

Analysis of mean FA and volume was performed using STATISTICA version 9.1 (2010; Stat Soft. Inc, Tulsa, OK, U.S.A.; http://www.statsoft.com). We used a repeated-measures analysis of variance to investigate group differences in regional volume and mean FA, including ROI (volume, mean FA) and side (left, right) as within-subject factors, and group (control, left TLEhs, and right TLEhs) as a between-subject factor. Multiple linear regression (R^2^) was used to examine relationships between structural volume and mean FA, and between ROIs and clinical variables. In investigating whether age of onset and duration of epilepsy correlated with brain architecture, all analyses were corrected for participant age in order to investigate the potential degenerative effects of epilepsy on volume and FA above the effects of normal aging. For correlational analyses in patients, various structures were treated as ipsilateral or contralateral to TLEhs in FA (hippocampus, parahippocampal gyrus, temporal lobe white matter, putamen and thalamus) and volume (hippocampus, temporal horn of the lateral ventricle, thalamus, putamen and cerebral cortex) analyses in order to generate increased statistical power. Callosal, frontal lobe white matter, brainstem, and internal capsule measures were treated bilaterally.

## Results

### A. Brain alterations in TLEhs relative to controls

#### (i) DTI alterations

Descriptive and inferential statistics for regional FA differences between patients and controls are provided in Table 2. Patients with left TLEhs had significantly reduced FA bilaterally in the hippocampus, parahippocampal gyrus, temporal lobe white matter, corpus callosum (including genu and splenium separately), frontal lobe white matter, internal capsule and brainstem. Conversely, patients with right TLEhs showed evidence of more restricted FA loss relative to controls, including the right parahippocampal gyrus, thalamus bilaterally, and the splenium/whole corpus callosum. Right hippocampal mean FA did not differ between patients with right TLEhs and controls. In order to investigate whether the restricted FA loss in right TLEhs was due to the decreased sample size (left TLEhs (n = 41), right TLEhs (n = 21)), we randomly reduced the number of patients with left TLEhs to n = 21, and found that all but one effect (right parahippocampal gyrus) were still highly significantly reduced in patients with left TLEhs (see Results S1).

**Table 2:** FA differences between controls (n = 68), patients with left MTS (n = 41) and patients with right MTS (n = 21).

FA-ROI	Controls		Left TLEhs				Right TLEhs			
	Mean	SD	Mean	SD	F	p	Mean	SD	F	p
**Hipp L**	122.45	(13.25)	114.81	(19.02)	6.076	0.02*	121.34	(12.75)	0.11	0.74
**Hipp R**	115.88	(13.73)	108.82	(13.07)	7.00	0.01**	117.33	(17.57)	0.16	0.69
**PHG L**	171.07	(16.60)	149.36	(21.30)	35.25	0.00000***	165.07	(12.26)	2.35	0.13
**PHG R**	171.08	(15.43)	164.26	(18.05)	4.40	0.04*	153.39	(13.54)	22.25	0.00001***
**TLWM L**	335.59	(22.42)	318.21	(22.41)	12.55	0.0006***	330.83	(20.73)	0.75	0.38
**TLWM R**	326.04	(24.12)	315.74	(27.70)	4.17	0.04*	317.10	(22.89)	2.26	0.14
**Thalamus L**	273.98	(22.52)	239.55	(35.64)	38.25	0.00000***	259.19	(21.94)	7.00	0.01**
**Thalamus R**	260.37	(17.57)	235.78	(3036)	28.74	0.00000***	243.14	(18.88)	14.9	0.0002***
**Putamen L**	162.64	(27.08)	171.26	(34.27)	2.12	0.15	161.59	(30.50)	0.02	0.88
**Putamen R**	172.04	(27.18)	172.51	(32.15)	0.007	0.94	161.68	(28.94)	2.26	0.14
**CC (W)**	400.71	(30.21)	371.51	(44.96)	16.44	0.0001***	382.87	(38.72)	4.88	0.03*
**CC (G)**	389.35	(35.29)	355.77	(48.08)	17.55	0.00006***	376.66	(44.73)	1.82	0.18
**CC (S)**	443.33	(30.15)	417.28	(44.64)	13.21	0.0004***	424.02	(32.53)	6.34	0.01**
**BFLWM**	322.61	(22.02)	308.60	(24.76)	9.42	0.003**	317.53	(25.96)	0.78	0.38
**Brainstem**	377.99	(19.94)	358.50	(27.70)	18.13	0.00004***	368.77	(19.28)	3.48	0.06
**BIC**	377.99	(19.94)	358.50	(27.70)	11.97	0.0008***	383.64	(20.77)	1.39	0.24

Note the more bilateral distribution of FA alterations in patients with left MTS. When the sample of patients with left MTS was randomly reduced to n = 21, all differences observed in the full sample remained highly significant with the exception of the right parahippocampal gyrus (F = 3.15, p = 0.07).

Significant at *p<0.05, **p<0.01, ***p<0.001, corrected for multiple comparisons.

Abbreviations: BFL, Bilateral Frontal Lobe White Matter; BIC, Bilateral Internal Capsule; CC, Corpus Callosum; G, Genu; Hipp, hippocampus; L, Left; PHG, parahippocampal gyrus; R, Right; S, Splenium; SD, Standard Deviation; TLWM, Temporal Lobe White Matter; W, Whole.

#### (ii) Volumetric alterations

**Figure 2:**
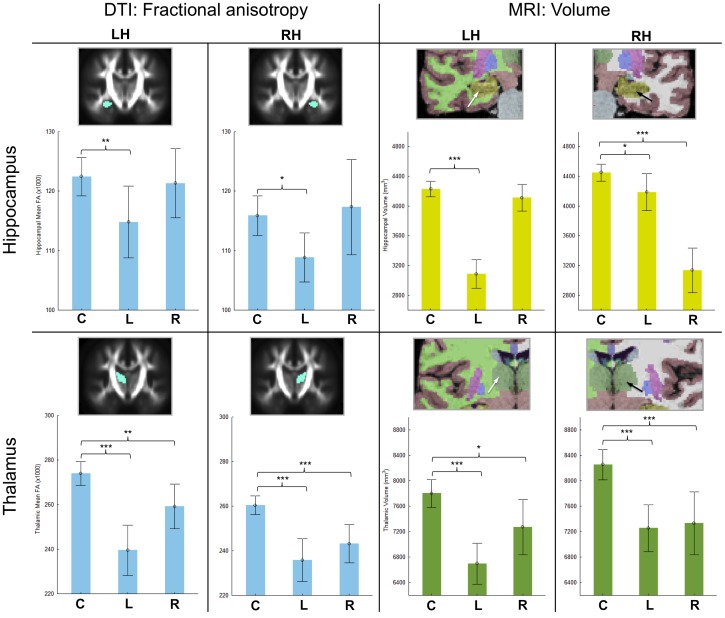
FA and volume alterations of the hippocampus and thalamus in patients with unilateral TLEhs relative to controls. The top row indicates mean (with 95% CI) FA (left) and volume (right) of the left and right hippocampus across controls (C), patients with left TLEhs (L) and patients with right TLEhs (R). The bottom row is the same for the thalamus. Structures are colour-coded: light blue for FA, yellow for hippocampal volume (as per standard FreeSurfer colour classification) and dark green for thalamic volume (as per standard FreeSurfer colour classification). FA values are the mean for each structure ×1000. Volumes are mm^3^. LH, left hemisphere. RH, right hemisphere. * = significant at p<0.05 (corrected). ** = significant at p<0.01 (corrected). *** = significant at p>0.001 (corrected).

Descriptive and inferential statistics of volume alterations in patients with left and right TLEhs relative to controls is presented in Table 3. Patients with left TLEhs had significant bilateral volume atrophy of the hippocampus (although, intuitively, much greater in the left hemisphere), thalamus, putamen, and cerebral cortex. All subregions other than the anterior-most segment of the corpus callosum were significantly atrophic. There was expansion of the left but not right temporal horn of the lateral ventricle in patients with left TLEhs relative to controls. Similarly to DTI results, patients with right TLEhs had more restrictive ipsilateral atrophy encompassing the right hippocampus, thalamus, putamen, and cortex, and expansion of the right temporal horn of the lateral ventricle. Only thalamic volume was significantly atrophic in the left hemisphere. Right TLEhs patients also had atrophy of the two posterior segments of the corpus callosum. As with analyses of FA, we reduced the number of patients with left TLEhs to n = 21 for auxiliary analyses of volume. Similarly to analyses of FA, only one structure did not remain significantly atrophic with the smaller sample size, the right hippocampus (see Results S1). Figure 2 shows a comparison of mean FA and volume of the hippocampus and thalamus across controls and patient groups in order to appreciate the concomitant alterations in T1-weighted MRI-derived and DTI-derived alterations of homologous brain regions.

**Table 3:** Volumetric differences between controls (n = 68), patients with left MTS (n = 41) and patients with right MTS (n = 21).

Volume of	Controls		Left TLEhs				Right TLEhs			
	Mean	SD	Mean	SD	F	p	Mean	SD	F	p
**Hipp L**	4228.9	(437.6)	3085.1	(602.2)	131.0	0.00000***	4110.6	(397.8)	1.22	0.27
**Hipp R**	4449.2	(469.40)	4186.6	(786.6)	4.78	0.03*	3136.1	(659.5)	102.6	0.00000***
**Thalamus L**	7799.8	(900.4)	6699.0	(1024.2)	34.45	0.00000***	7272.5	(949.3)	5.37	0.02*
**Thalamus R**	8251.7	(971.9)	7254.9	(1162.6)	23.18	0.00001***	7330.8	(1078.6)	13.69	0.0004***
**Putamen L**	5371.0	(723.7)	4737.2	(714.2)	19.81	0.00002***	5016.9	(683.3)	3.94	0.06
**Putamen L**	5369.1	(715.5)	4870.0	(705.8)	12.57	0.0006***	5015.9	(608.3)	4.18	0.04*
**THLV L**	216.5	(163.8)	415.1	(280.5)	21.84	0.00001***	189.1	(153.0)	0.46	0.49
**THLV R**	338.1	(240.9)	349.6	(209.3)	0.06	0.80	536.4	(319.4)	9.26	0.003**
**Cortex L**	253320.2	(26630.7)	226884.7	(31825.6)	21.73	0.00001***	247192.0	(28329.0)	0.83	0.37
**Cortex R**	254876.2	(26576.0)	232490.2	(32779.9)	15.19	0.0002***	241236.6	(27616.3)	4.15	0.04*
**CC (P)**	904.7	(126.6)	842.8	(199.4)	3.94	0.05*	832.0	(119.6)	5.42	0.02*
**CC (MP)**	467.6	(92.0)	380.8	(104.0)	20.64	0.00002***	410.0	(90.6)	6.35	0.01**
**CC (Cen)**	470.0	(96.2)	396.5	(98.3)	14.66	0.0002***	446.5	(94.2)	0.96	0.32
**CC (MA)**	500.6	(113.9)	415.3	(108.4)	14.87	0.0002***	474.3	(100.4)	0.90	0.34
**CC (A)**	881.3	(143.7)	825.2	(148.2)	3.80	0.054	873.5	(142.9)	0.05	0.83

Note the more bilateral distribution of atrophy in patients with left MTS. When the sample of patients with left MTS was randomly reduced to n = 21, all differences observed in the full sample remained highly significant with the exception of the right hippocampus (F = 1.30, p = 0.26).

Significant at *p<0.05, **p<0.01, ***p<0.001, corrected for multiple comparisons.

Abbreviations: A, Anterior; CC, corpus callosum; Cen, Central; Hipp, hippocampus; L, Left; MA, Middle Anterior; MP, Middle Posterior; P, Posterior; R, Right; SD, Standard Deviation; THLV, Temporal Horn of Lateral Ventricle.

### B. Direct comparison between left and right TLEhs

**Figure 3:**
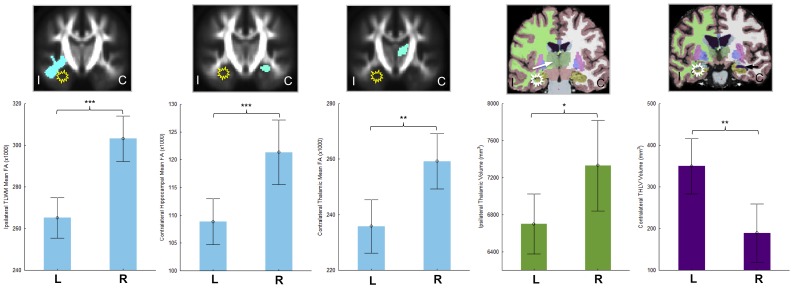
Significantly greater FA and volume alterations in patients with left TLEhs relative to right TLEhs. Mean and 95% CI are shown. Structures are illustrated ipsilateral or contralateral to the medial temporal epileptogenic focus (yellow/white star). From left to right; ipsilateral thalamic FA, contralateral hippocampal FA, contralateral thalamic FA, ipsilateral thalamic volume, and contralateral temporal horn of the lateral ventricle volume. Colour coding is the same as in Figure 2. Purple is temporal horn of the lateral ventricle volume (as per standard FreeSurfer colour classification). FA values are the mean for each structure ×1000. Volumes are mm^3^. C, contralateral; I, ipsilateral; , left TLEhs; R, right TLEhs. * = significant at p<0.05 (corrected). ** = significant at p<0.01 (corrected). *** = significant at p>0.001 (corrected).

Relative to controls, patients with left TLEhs showed a greater degree and more bilateral distribution of FA and volumetric reduction compared to patients with right TLEhs. In order to directly examine the differences in brain alterations between left and right TLEhs, we compared FA and volumetric measures of structures ipsilateral and contralateral to TLEhs between patient groups. Of all anatomical variables investigated, there were five statistically significant differences between patients with left and right TLEhs. Patients with left TLEhs had significantly decreased ipsilateral thalamic volumes (F = 5.10, p = 0.03) and temporal lobe white matter FA (F = 24.49, p<0.001), decreased contralateral hippocampal (F = 12.94, p = 0.001) and thalamic (F = 9.82, p = 0.003) FA, and increased expansion of the contralateral temporal horn of the lateral ventricle (F = 9.66, p = 0.003) relative to patients with right TLE. These differences are shown in Figure 3. All significant differences persisted when the sample of patients with left TLEhs were reduced in number to match that of patients with right TLEhs. Patients with right TLEhs did not exhibit volume or FA reduction greater than patients with left TLEhs in any region examined.

### C. DTI-atrophy relationships

**Figure 4:**
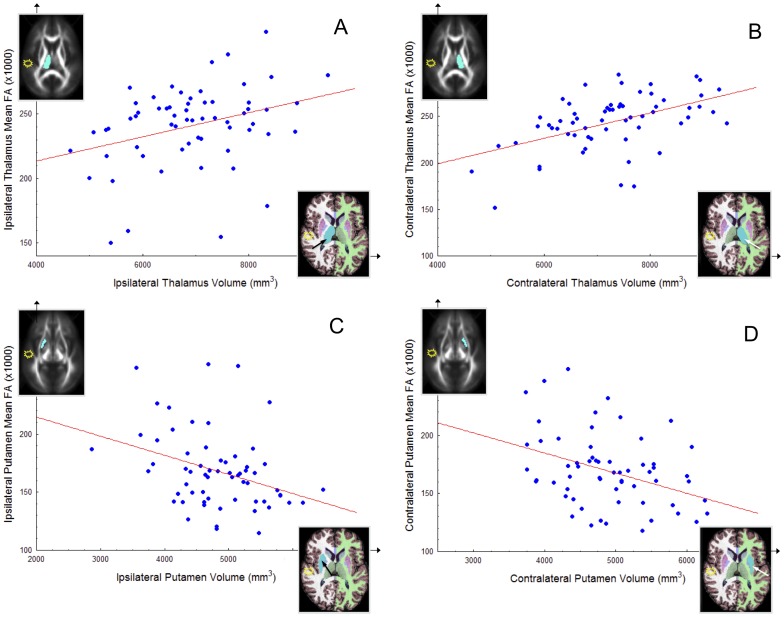
Direct significant correlations between FA and volume in patients with unilateral TLEhs. (A) Ipsilateral thalamus. (B) Contralateral thalamus. (C) Ipsilateral putamen. (D) Contralateral putamen. Structures are illustrated ipsilateral or contralateral to the medial temporal epileptogenic focus (yellow/white star). FA values are the mean for each structure ×1000.

We investigated the relationship between volume and FA of homologous subcortical structures (hippocampus, thalamus, putamen and corpus callosum) and temporal lobe white matter FA ipsilateral and contralateral to TLEhs. Significant correlations indicative of concomitant volume atrophy and FA loss were observed in the contralateral hippocampus (r = .28, p = 0.03), ipsilateral thalamus (r = .33, p = 0.01) and contralateral thalamus (r = .50, p<0.001). The volume of the contralateral hippocampus was also significantly positively correlated with the FA of the contralateral temporal lobe white matter (r = .31, p = 0.02). No significant relationship was found between volume and FA of the ipsilateral hippocampus (r = .09, p = 0.50). There were significant negative correlations indicative of decreasing volume and increasing FA of the ipsilateral (r = −.35, p = 0.006) and contralateral (r = −.38, p = 0.002) putamen. Significant associations between volume and FA remained when patients were separated according to side of TLEhs. The relationships between volume and FA of the thalamus and putamen are shown in Figure 4.

### D. Correlations with clinical data

**Figure 5:**
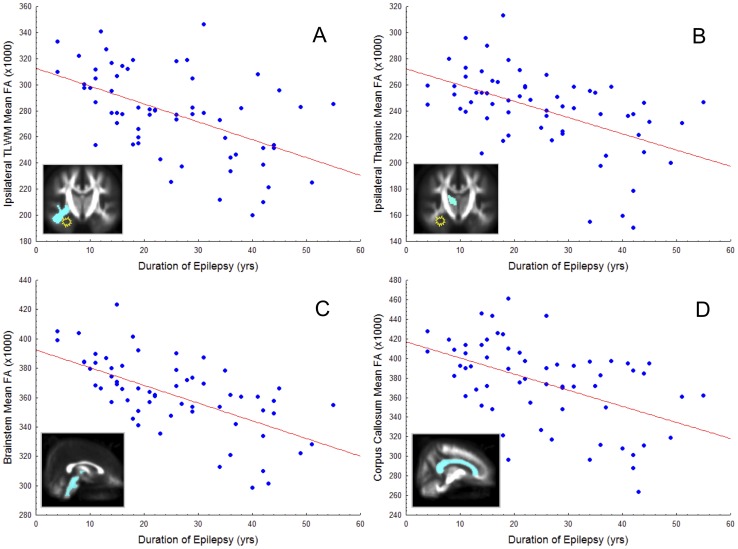
Selected significant correlations between regional mean FA (×1000) and duration of epilepsy (years), corrected for age. Unilateral structures are illustrated ipsilateral or contralateral to the medial temporal epileptogenic focus (yellow/white star). A. Ipsilateral temporal lobe white matter. B. Ipsilateral thalamus. C. Brainstem. D. Whole corpus callosum.

**Figure 6:**
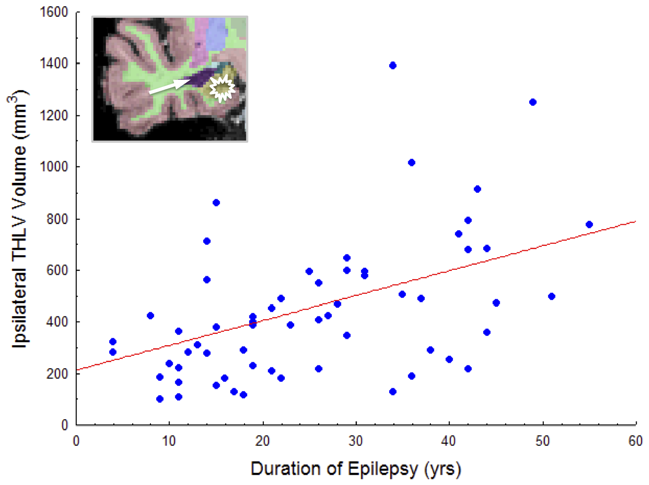
Significant correlation between volume (mm^3^) expansion of the ipsilateral temporal horn of the lateral ventricle and duration of epilepsy in patients with unilateral TLEhs, corrected for age.

When controlled for age, duration of epilepsy was significantly negatively correlated with mean FA of the ipsilateral temporal lobe (r = −.526, p<0.001), ipsilateral parahippocampal gyrus (r = −.324, p = 0.04), ipsilateral thalamus (r = −.529, p<0.001), contralateral thalamus (r = −.547, p<0.001), corpus callosum (r = −.500, p<0.001), splenium (r = −.452, p = 0.001), internal capsule (r = −.629, p<0.001), and brainstem (r = −.639, p<0.001) in all patients (see Figure 5 for selected illustrations). All other relationships were not significant when corrected for age and multiple comparisons (p>0.05). Age of onset of epilepsy was not significantly correlated with any FA measure. The only clinical-volume correlation observed (corrected for age) was between duration of epilepsy and ipsilateral temporal horn of the lateral ventricle (r = .453, p = 0.004), manifested as increasing expansion with increasing duration. This relationship is shown in Figure 6. See Results S1 for age-FA correlations. Patients with a history of febrile convulsions did not differ in any ipsilateral or contralateral DTI or volumetric value compared to patients without such a history.

## Discussion

Using multi-modal MRI and DTI to investigate gross morphological and (a proxy for) microarchitectural alterations in a relatively large sample of patients with unilateral TLEhs, we report (i) preferential ipsilateral temporal lobe and bilateral extratemporal lobe FA and volume alterations in patients with unilateral TLEhs, (ii) that volumetric atrophy is associated with alterations in FA in homologous structures, (iii) patients with left TLEhs have a more widespread and bilateral distribution of brain alterations relative to their right-sided counterparts, and (iv) duration of epilepsy is significantly correlated with FA loss of ipsilateral temporal lobe structures, the thalamus bilaterally, and medial structures including the corpus callosum, brainstem, and internal capsule. The sole clinical-anatomical correlation obtained from morphometric analysis of MRI was between increasing duration of epilepsy and expansion of the ipsilateral temporal horn of the lateral ventricle. A discussion of pertinent methodological issues is provided prior to a discussion of the biological interpretations of this data.

### Methodological issues

Whilst combined ipsilateral hippocampal volume atrophy and FA loss was observed in patients with left TLEhs, only hippocampal volume atrophy was observed in patients with right TLEhs. Patients with right TLEhs did not show any significant difference in ipsilateral hippocampal FA relative to healthy controls, despite showing strong and significant ipsilateral parahippocampal FA loss. We are unsure as to why this is the case. It may have been conceived that the hippocampal mask used for quantification of mean FA being was too liberal to accurately quantify FA in hippocampal tissue per se, and may have included other illegitimate tissue in patients with altered medial temporal lobe structure (i.e. that hippocampal shrinkage and resultant displacement of the stereotaxic mask may have caused sampling of the low-FA temporal horn of the lateral ventricle within the hippocampal mask). However, the right hippocampal mask was generated in the exact same fashion as the left hippocampal mask, and patients with left TLEhs showed significant hippocampal FA loss in the main sample, and the sample size-matched with patients with right TLEhs. We furthermore cannot attribute the differences in hippocampal FA between patient groups to a greater degree of hippocampal damage in patients with left TLEhs given the non-significant difference in ipsilateral hippocampal FA between patient groups (3085 mm3 left TLEhs, 3136 mm3 right TLEhs; p = 0.76). Patients with right TLEhs did have a greater variance of ipsilateral hippocampal FA values compared to controls and patients with left TLEhs (see Figure 2 and Table 2), but this variance could not be explained by outlying right hippocampal FA values. It is also noted that a volume-FA correlation of the sclerotic hippocampus was not observed in this study. This is likely due to the small variance in patient hippocampal metrics given that the primary inclusion criterion for the enrolment of patients into this study was clear unilateral hippocampal sclerosis. Therefore, analysis of hippocampal volume-FA relationships may be observed in a sample of patients not prospectively enrolled according to clear hippocampal structural pathology. This is supported by the finding of a significant correlation between volume and FA for the hippocampus contralateral to hippocampal sclerosis where the variance of hippocampal metrics is much greater (i.e. not all pathological values).

Cross-sectional analyses of the clinical variables hypothesized to be related to brain degeneration are complicated by the subjective recollection of the patient and patient family members' of the occurrence of the initial seizure. Prior to this there is also the strong chance that aberrant neurological events may have caused undetected brain damage. In particular, early childhood febrile convulsions and other initial precipitating injuries can be followed by a long latent period prior to the onset of habitual epilepsy, with the early damage potentially causing hippocampal and, theoretically, extrahippocampal brain damage. We found no evidence of increased hippocampal or extrahippocampal damage in patients with a history of childhood febrile convulsions in the present study. This is consistent with previous work with respect to extrahippocampal brain structure [17], but inconsistent with work showing increased hippocampal atrophy in patients with such a history [17], [36], [37]. This discrepancy is likely to be due to the fact that only patients with clear hippocampal sclerosis were enrolled in this study, unlike previous studies that were able to investigate a wider range of hippocampal volumes. The correlations between FA degeneration and a longer duration of TLEhs - corrected for the effects of normal aging - reported in this manuscript are clinically and anatomically plausible (see below), and are *suggestive* of progressive brain damage in response to insufficiently controlled recurrent seizures and/or chronic use of antiepileptic medication. However, these findings must be confirmed using longitudinal imaging studies, particularly in studies where baseline imaging is performed at the time of epilepsy diagnosis. Given that DTI can reveal pathological alterations in brain integrity beyond the resolution of conventional MRI - and that these alterations may have a real biological basis [11] - it is not surprising that brain-clinical correlations were almost exclusively observed using DTI.

We quantified regional volume and FA using automated approaches. With respect to volume estimation, automated methods are particularly advantageous in large cohort studies given the time required to generate volumes for individual brain structures by an expert anatomist using ROI manual approaches. However, automated methods require validation with respect to manual measures. There is a growing amount of literature demonstrating consistency between automated FreeSurfer and manual measures of subcortical volume [33], [34], [35]. Data presented in this paper inherently provides evidence for the reliability of automated hippocampal volume estimation in patients with neurological disorders, given that hippocampal sclerosis was identified prior to the enrolment of patients into this study. Indeed, when comparing the left and right hemispheres of the randomly selected patient with right TLEhs shown in Figure 1, the excellent automated labelling of the sclerotic hippocampus and the associated expansion of the ipsilateral temporal horn of the lateral ventricle can be clearly visualised.

Given that axonal membranes play the primary role for significant anisotropy in DTI data [38], the study of FA has largely been limited to that of white matter. Furthermore, noise in DTI data can bias the calculated diffusion tensor and lead to an overestimate of diffusion anisotropy when the signal-to-noise ratio and actual anisotropy are low [39], [40], which may potentially overwhelm FA in areas with relatively low diffusion anisotropy such as gray matter [41]. However, DTI analysis of gray matter nuclei may have an architecturally and physiologically meaningful basis in epilepsy. Focal FA abnormalities of deep gray matter structures have been reported in patients with epilepsy, including structures with the lowest FA values such as the putamen [42], [43], [44], [45]. These alterations have been shown to correlate with duration and age of onset of a disorder [45], with macroscopic volume of the same structure [44], [45], and progressively alter in response to recurrent seizures [43]. Furthermore, FA alterations of the striatum has been demonstrated in patients with known disorders of these nuclei [46], [47], and striatal FA changes have been demonstrated in response to increased iron accumulation in normal aging [48]. Increasing FA has been also demonstrated in even smaller deep gray matter structures including the globus pallidus and substantial nigra in Hallervorden-Spatz disease, reflecting a pathological accumulation of iron concentration in these structures [49]. Importantly, several studies have reported reduction of hippocampal FA in patients with TLE [50], [51], [52], indicating that quantitative analysis of FA of the hippocampus reveals diffusion-based pathology underlying/associated with epileptiform activity, despite the low FA values of subcortical gray matter. Similarly, significant alterations of thalamic FA are a frequent finding in multiple epilepsy syndromes [7], [44], [53], [54]. DTI-derived pathological thalamic measures have previously been attributed to structural disorganization and the expansion of extra-cellular space resulting from the noxious effects of epileptiform activity [42].

### Biological interpretations

The distribution of T1-weighted MRI and DTI determined brain abnormalities in patients with TLEhs - and indeed, many neurological disorders - are typically independently disseminated in different groups of patients. For example, structural atrophy of the thalamus has previously been reported in patients with TLEhs [1], [6], and thalamic diffusion alterations have been reported in other studies [20], [51]. The same is true for the hippocampus in many more studies. To our knowledge, there are no studies that have directly investigated the relationship between morphological atrophy and FA alterations of homologous brain structures in the same patients with TLEhs, which is an approach that is best suited to gain a more detailed understanding of macroscopic-microscopic pathological brain alterations *in vivo*. Our data indicates that hippocampal sclerosis and extrahippocampal atrophy in patients with TLEhs are reflected by concomitant volume loss, a proxy for neuronal atrophy, and FA loss, a proxy for altered microstructural organisation. This relationship is particularly evident in the thalamus, which exhibits a strong relationship between bilateral atrophy and FA loss in unilateral patients. The relationships in the contralateral temporal lobe are interesting, and may be indicative of a pathological process. Abnormalities of the hippocampus [55], [56] and extrahippocampal temporal lobe [1], [57] contralateral to the epileptogenic focus are sometimes reported in patients with unilateral TLEhs. Unlike the thalamus and temporal lobe structures, volume-FA relationships in the putamen were inverted in patients, manifested as significantly related volume atrophy and pathological FA increase. This effect has been recently reported in patients with MRI-negative epilepsy syndromes [8], [9]. The mechanisms behind this inverted relationship are not well understood, but may include (i) a direct affect of grey matter atrophy on the sampling density of myelinated fibres within the putamen or (ii) increasing putamen iron concentration in response to aberrant neurological events, as discussed in more detail previously [8], [9].

#### Progressive neurologic injury in TLE?

We cannot disambiguate whether the primary factor causing accelerated FA loss in patients with TLEhs is due to a degenerative effect of recurrent seizures or the chronic use of antiepileptic medication. However, it is clear from our data that the effects of having TLEhs are related to brain degeneration that is accelerated beyond the effects of normal aging. Other longitudinal and cross-sectional papers based on T1-weighted MRI have reported mixed findings. Some longitudinal studies have revealed subtle hippocampal [58], [59] and extrahippocampal [12] alterations over a relatively short period of time. Conversely, other longitudinal studies have found no evidence of progressive seizure-induced atrophy superseding normal age-related atrophy [60], [61]. Some cross-sectional studies have reported correlations between duration of TLE and various metrics (e.g., volume, cortical thickness) of the hippocampus [18], [62], temporal lobe [18], [63], subcortical structures [17], [18], [64], and extratemporal cortex [17], [18], [65], suggestive of structural alterations in response to epileptic seizures or chronic use of anticonvulsant therapy. However, several studies have reported no correlations between clinical information and structural measures from T1-weighted MRI in patients with TLEhs [6], [19], [66], [67]. It may be expected that associations between regional brain architecture and clinical variables would be more revealing using DTI, given the increased sensitivity of DTI over conventional MRI in the detection of pathological brain alterations [7], [38], which is supported by the findings presented in this paper. In particular, we found evidence of widespread regional FA-duration of epilepsy correlations, including the necessary correction for age, but only one volumetric-duration correlation. Other cross-sectional DTI studies investigating such associations have used substantially reduced sample sizes relative to the present study, which may explain the frequent finding of no or minimal statistically significant correlations between DTI measures and duration of epilepsy. These studies have included samples of eight [68], nine [20], 12 [69], 15 [70], and 35 [4] patients with TLEhs.

The topological distribution of FA degeneration related to the duration of epilepsy as reported in the present study follows an anatomically plausible route, including the ipsilateral parahippocampal gyrus, ipsilateral temporal lobe white matter, and primarily posterior regions of the corpus callosum (as reflected in the findings of the splenium) that consist of inter-hemispheric temporal lobe fibres [71]. Duration-FA relationships of the internal capsule and brainstem are suggestive of a compromise of white matter integrity of corticospinal tracts in response to TLEhs. The duration-volume relationship of the ipsilateral temporal horn of the lateral ventricle also exceeded that seen in normal aging, which may be reflection of continuing medial temporal lobe atrophy (although note no significant correlation between ipsilateral hippocampal volume and duration of TLEhs, and therefore may involve other neuropathological processes). The results reported here are consistent with a previous study that reported no correlation between duration of epilepsy and ipsilateral hippocampal volume, but did with respect to the thalamus bilaterally [17]. Hippocampal sclerosis may be a progressive abnormality [58], [59], but it is likely that significant hippocampal damage may occur in response to initial precipitating factors that may trigger neuropathological processes that later cause habitual chronic epilepsy [14]. Importantly, the lack of hippocampal (FA or volume)-duration of epilepsy correlations reported in this manuscript (and indeed, in Keller et al. [17] previously) may be due to the enrolment of patients with well-characterized hippocampal sclerosis, so that a wide range of patient hippocampal metrics - which would be needed to reveal significant correlations - were not incorporated into analysis (as discussed above).

Longitudinal and cross-sectional neuropsychological assessment has been used to infer progressive neurologic injury in patients with epilepsy. A recent longitudinal study reported progressive deterioration in an array of cognitive abilities between assessment at the time of epilepsy diagnosis and follow up at one year, demonstrating a different cognitive trajectory in patients relative to healthy controls [72]. Progressive deterioration of memory functions has also been demonstrated in patients with chronic TLE in another longitudinal study [73]. This study also reported that memory decline may cease and even normalize when seizures are fully controlled [73]. Significant correlations between increasing duration of epilepsy and declining neuropsychological performance has been reported in several cross-sectional studies [74], [75], [76], [77], [78], which are suggestive of degenerative neurological processes in TLEhs. However, there is evidence for neuropsychological impairment at the time of epilepsy diagnosis with minimal progressive deterioration thereafter that exceeds what is observed in normal aging [79]. There is evidence suggesting that verbal memory deficits are present in patients with newly diagnosed epilepsy and are of similar extent to patients with uncontrolled TLE of 30 years [80]. This same study indicated that verbal memory does not further deteriorate five years after baseline [80]. Other studies have reported no progressive deterioration in neuropsychological performance, seizure frequency, seizure severity, and electroencephalographic patterns after years of patient follow up [81], [82], [83]. Such evidence suggests that brain damage causing neuropsychological impairments may precede epilepsy diagnosis. Ultimately, only combined DTI, functional imaging and neuropsychological assessment of patients at the point of epilepsy diagnosis with longitudinal follow up at various time intervals will convincingly disentangle whether recurrent seizures have a noxious and degenerative effect on brain structure and function.

#### Side of TLEhs matters

Consistent with other studies [22], [26], [27], [28], we reported a more bilateral distribution of brain alterations in patients with left TLEhs compared to right TLEhs. Not only were these findings shown relative to controls, but also in direct comparisons between patient groups, in unequal and equal group sizes. These findings reinforce the notion that left and right TLEhs can be viewed as aetiologically distinct and pathologically different [27]. One can only speculate as to why patients with left and right TLEhs have different patterns of brain alterations. It may be that neuronal connections in the left hemisphere are more likely to support seizure propagation to the contralateral hemisphere [27]. As in the present study, previous work has shown that differences in anatomical alterations between patients with left and right TLEhs cannot be attributed to clinical variables, such as differences in duration of epilepsy, age of onset of epilepsy, seizure frequency, history of childhood febrile convulsions, or antiepileptic medication exposure [22]. It is therefore likely that the aetiology of differences in brain degeneration between patients with left and right TLEhs is based on a combination of architectural, connectivity, physiological and developmental differences between the hemispheres. Regardless of the causes of these differences, patients with left TLEhs consistently perform worse on neuropsychological tests that do not solely rely on the ipsilateral temporal lobe or even the ipsilateral cerebral hemisphere relative to their right-sided counterparts [84]. This may not necessarily be explained by lateralized damage to the language-dominant hemisphere, but perhaps by the fact that left TLEhs is associated with a diffuse and bilateral distribution of grey and white matter degeneration, whereas right TLEhs is associated with more restricted ipsilateral alterations. Importantly, the results presented in this paper indicate that patients with left TLEhs have significant reduction of frontal lobe white matter mean FA relative to controls, whereas right TLEhs patients did not (Table 2). This finding corroborates other work reporting increased frontal lobe vulnerability in patients with left TLEhs relative to right TLEhs [17], [22]. It is therefore possible that increased frontal lobe degeneration, and extra-temporal lobe degeneration more generally, in patients with left TLEhs may explain the exacerbated cognitive impairments seen in these patients relative to their right-sided counterparts [28]. Perhaps contrary to expectation in light of increased pathological brain alterations in left TLEhs, patients with right TLEhs have significantly lower post-surgical remission and higher mortality rates compared to patients with left TLEhs [85]. The neuropathological factors underlying this outcome are as yet undetermined.

### Conclusion

TLEhs is associated with interrelated DTI-derived and T1-weighted MRI-derived brain degenerative abnormalities that are influenced by the duration of the disorder and the side of seizure onset. True clinical progress in the understanding of the progression of anatomical alterations in response to recurrent seizures can only be achieved using longitudinal DTI studies with simultaneous consideration of individual biological and clinical variables, preferably starting at the time of epilepsy diagnosis. However, the cross-sectional data presented here suggests that TLEhs is a neurodegenerative disorder, although the underlying mechanisms causing brain alterations may be different from classical neurodegenerative disorders.

## Supporting Information

Results S1 (A) DTI and volumetric differences between patients and controls using matched sample sizes. (B) Correlations between FA and age in patients and controls.
(DOC)
