# Supplementary material for: Concomitant Fractional Anisotropy and Volumetric Abnormalities in Temporal Lobe Epilepsy: Cross-Sectional Evidence for Progressive Neurologic Injury
Source: PLoS One. 2012 Oct 11;7(10):e46791. doi: 10.1371/journal.pone.0046791 (PMC3469561; doi:10.1371/journal.pone.0046791)
Supplement: Results S1 — (A) DTI and volumetric differences between patients and controls using matched sample sizes. (B) Correlations between FA and age in patients and controls. (DOC) [file pone.0046791.s001.doc]

Concomitant microstructural and volumetric brain abnormalities in

temporal lobe epilepsy

**Supplementary Results**

A. Brain alterations in TLEhs relative to controls: results for patients with left TLEhs (n=21)

*(i) DTI alterations*

Using the reduced sample size of patients with left TLEhs (n=21), left (F=8.76, p=0.004) and right (F=4.31, p=0.04) hippocampal, left parahippocampal (F=32.91, p<0.001), left (F=18.43, p<0.001) and right (F=5.08, p=0.03) temporal lobe white matter, left (F=33.41, p<0.001) and right (F=26.94, p<0.001) thalamic, whole corpus callosum (F=14.50, p=0.001), genu (F=11.28, p=0.001), splenium (F=15.16, p<0.001), frontal lobe white matter (F=7.58, p=0.007), internal capsule (F=11.76, p=0.001), and brainstem (F=12.98, p=0.001) mean FA was significantly reduced in patients relative to controls. Only right parahippocampal FA did not remain statistically significantly different between patients with left TLEhs and controls (F=3.15, p=0.08) when the sample size was reduced.

*(ii) Volumetric alterations*

Using the reduced sample size of patients with left TLEhs (n=21), the left hippocampus (F=100.45, p<0.001), left (F=24.65, p<0.001) and right (F=14.01, p=0.001) thalamus, left (F=13.61, p=0.001) and right (F=7.92, p=0.006) putamen, left (F=, p=0.001) and right (F=, p=0.02) cerebral cortex, posterior (F=5.36, p=0.03), mid posterior (F=16.60, p<0.001), central (F=10.35, p=0.002), and mid anterior (F=7.60, p=0.007) corpus callosum were significantly reduced in volume in patients relative to controls. Only right hippocampal volume did not remain different between patients with left TLEhs and controls (F=1.30, p=0.26) compared to the full sample. The volume of the left temporal horn of the lateral ventricle remained significantly increased in patients (F=29.54, p<0.001).

B. FA-age correlations

We investigated the expected relationships between regional FA and age in patients and controls. These results are shown in Supplementary Table 1 (below) and indicate that the FA of all brain regions examined significantly correlated with age in controls after correction for multiple comparisons. Whilst there were also significant relationships between regional FA and age in patients, there were areas that did not significantly correlate, including the hippocampus and splenium. For all regions other than the left and right putamen, correlations indicated relationships between increasing age and reduced FA. Putamen FA significantly increased with age.

| **FA-ROI** | **Controls** | | **Left TLEhs** | | **Right TLEhs** | | **All patients** | |
| --- | --- | --- | --- | --- | --- | --- | --- | --- |
|  | **r** | **p** | **r** | **p** | **r** | **p** | **r** | **p** |
| **Hipp L** | -.388 | 0.001 | -.259 | 0.10 | -.114 | 0.62 | -.225 | 0.08 |
| **Hipp R** | -.307 | 0.01 | -.345 | 0.03 | -.032 | 0.89 | -.200 | 0.12 |
| **PHG L** | -.422 | 0.0003 | -.090 | 0.58 | -.209 | 0.36 | -.143 | 0.27 |
| **PHG R** | -.435 | 0.0002 | -.397 | 0.01 | -.250 | 0.28 | -.300 | 0.01 |
| **TLWM L** | -.564 | <0.0001 | -.435 | 0.004 | -.403 | 0.07 | -.426 | 0.001 |
| **TLWM R** | -.606 | <0.0001 | -.473 | 0.002 | -.254 | 0.27 | -.397 | 0.001 |
| **Thalamus L** | -.287 | 0.02 | -.435 | 0.005 | -.532 | 0.01 | -.451 | <0.0001 |
| **Thalamus R** | -.287 | 0.02 | -.400 | 0.01 | -.385 | 0.08 | -.386 | 0.002 |
| **Putamen L** | .534, | <0.0001 | .389 | 0.01 | .346 | 0.12 | .380 | 0.002 |
| **Putamen R** | .476 | <0.0001 | .492 | 0.001 | .149 | 0.52 | .381 | 0.002 |
| **CC (W)** | -.475 | <0.0001 | -.335 | 0.03 | -.433 | 0.05 | -.372 | 0.003 |
| **CC (G)** | -.498 | <0.0001 | -.422 | 0.006 | -.501 | 0.02 | -.501 | 0.02 |
| **CC (S)** | .507 | <0.0001 | -.293 | 0.06 | -.328 | 0.15 | -.328 | 0.15 |
| **BLFLWM** | .607 | <0.0001 | -.487 | 0.001 | -.574 | 0.006 | -.526 | <0.0001 |
| **Brainstem** | .624 | <0.0001 | -.493 | 0.001 | -.643 | 0.002 | -.529 | <0.0001 |
| **BIC** | .601 | <0.0001 | -.467 | 0.002 | -.589 | 0.005 | -.588 | 0.005 |

**Supplementary Table 1.** Correlations between regional FA and age in controls and patients (corrected for multiple comparisons).

The FA in all white matter regions, hippocampus, parahippocampal gyrus, thalamus and brainstem declines with increasing age. Putamen FA increases with age.

Abbreviations: BFL, Bilateral Frontal Lobe White Matter; BIC, Bilateral Internal Capsule; CC, Corpus Callosum; G, Genu; Hipp, hippocampus; L, Left; PHG, parahippocampal gyrus; R, Right; S, Splenium; TLWM, Temporal Lobe White Matter; W, Whole.
